# Application of the 2013 ACR/EULAR classification criteria for systemic sclerosis to patients with Raynaud’s phenomenon

**DOI:** 10.1186/s13075-015-0594-5

**Published:** 2015-03-22

**Authors:** Jin-Su Park, Min-Chan Park, Jason Jungsik Song, Yong-Beom Park, Soo-Kon Lee, Sang-Won Lee

**Affiliations:** Division of Rheumatology, Department of Internal Medicine, Yonsei University College of Medicine, 50 Yonsei-ro, Seodaemun-gu, Seoul 120-752 South Korea

## Abstract

**Introduction:**

We investigated how many patients, who presented with Raynaud’s phenomenon (RP) and who had not been classified as systemic sclerosis (SSc), would be reclassified as SSc, if the 2013 American College of Rheumatology (ACR)/the European League Against Rheumatism (EULAR) classification criteria were used. We also analyzed the predictive values of the reclassification as SSc in those patients.

**Methods:**

We consecutively enrolled 64 patients with RP and 60 patients with SSc. We applied the new classification criteria to them, reclassified them, and compared variables between those who were newly classified as SSc and those who were not or previously classified as SSc.

**Results:**

Seventeen of 64 patients (26.5%), who presented with RP, but did not fulfill the 1980 ACR classification criteria, were newly classified as SSc by the 2013 ACR/EULAR classification criteria. The newly classified patients as SSc showed increased frequencies of sclerodactyly, digital tip ulcer, telangiectasia, abnormal nailfold capillaries and the presence of anti-centromere antibody, compared to those not and telangiectasia and anti-centromere antibody, compared to the previously classified patients. For the reclassification as SSc, the variables with independent predictive value were sclerodactyly (odds ratio (OR) 60.025), telangiectasia (OR 13.353) and the presence of anti-centromere antibody (OR 11.168).

**Conclusions:**

Overall, 26.5% of the patients, who presented with RP, but who did not fulfill the 1980 ACR classification criteria, were newly classified as SSc according to the 2013 ACR/EULAR classification criteria. Sclerodactyly, telangiectasia, and the presence of anti-centromere antibody had independent predictive value for reclassifying patients with RP as SSc.

## Introduction

Raynaud’s phenomenon (RP) is a reversible vasculopathy characterized by pallor and cyanosis due to cold-induced paroxysmal spasms of the digital vessels and hyperemia in the recovery phase [1]. It is not a very rare symptom and its prevalence ranges from 3% to 5% [2]. RP can be classified as Raynaud’s syndrome, when there is an associated disease. Meanwhile, it can be referred to as Raynaud’s disease, when there is no clear aetiology [3]. RP may be mediated and aggravated by three mechanisms: (i) neurogenic disorders, (ii) inadequate interaction between blood and vessel walls, (iii) changes in immune regulation [3]. Among immune-mediated conditions affecting the development of RP, systemic sclerosis (SSc) is the most common, representing 85% of such cases [4].

SSc is a systemic autoimmune disease characterized by vasculopathy and fibrosis of both the skin and internal organs, with pulmonary arterial hypertension, interstitial lung disease and gastrointestinal manifestations [5-8]. The typical pathological features of SSc are mostly progressive and irreversible, so it could often reduce one’s quality of life and be fatal enough to shorten life expectancy [9]. Thus, there is a need to slow down the progression of SSc, but the efficacies of several therapeutic modalities, that have been assessed in clinical trials, have not yet been fully validated. Considering the intractable and serious systemic complications of SSc and the lack of established predictive values for their development, we expect that early classification of SSc, if possible, would provide better opportunity to monitor its development or progression. From a clinical point of view, RP might be a valuable clue prior to the initiation of SSc for the following reasons: (i) SSc is the most common immune-mediated aetiology of RP, (ii) RP is a clinical feature frequently observed in the early phase of SSc, (iii) RP can often precede skin and visceral fibrosis in SSc [4]. Nevertheless, the 1980 American College of Rheumatology (ACR) classification criteria for SSc did not include an item for RP [10].

More recently, the ACR and the European League Against Rheumatism (EULAR) recommended the new classification criteria for SSc [11]. Their new classification criteria have a notable feature in that they include new variables on clinical features in the early phases of the disease such as RP, puffy finger, and telangiectasia, and the test results of capillary microscopy and autoantibody tests [11]. With these changes, they raised the sensitivity of classification by up to approximately 15 to 20%, compared to the previous classification criteria.

Thus, in this study, we investigated how many patients, who presented with RP and who had not been classified as SSc, would be reclassified as SSc, if the 2013 ACR/EULAR classification criteria were used. We also analyzed the predictive values of the reclassification as SSc in those patients.

## Methods

### Patients

We consecutively enrolled 64 patients (58 women, 6 men) with RP who had not been classified as SSc by the 1980 ACR classification criteria, and who had been referred to the Division of Rheumatology, Yonsei University Severance Hospital, from November 2013 to May 2014 for evaluating the underlying causes of their RP. We applied the 2013 ACR/EULAR classification criteria to 64 patients with RP, and we further assessed several classification criteria for various rheumatic diseases that can cause RP or RP-like symptoms. We also consecutively enrolled 60 patients (52 women, 8 men) who had been classified as SSc at the same institution, and who visited our division during the same time period. We applied the 2013 ACR/EULAR classification criteria for SSc to both RP and SSc patients as classified by the 1980 ACR classification criteria. Patients with a total score of ≥9 were reclassified as SSc [11]. After the reclassification, we compared variables between those who were newly classified as SSc and those who were not. We also assessed the predictive value of the reclassification for variables that significantly differed between the groups. In addition, we compared variables between patients who were newly classified as SSc versus those who had been classified as SSc by the previous criteria. This study was approved by the Institutional Review Board of Severance Hospital. Informed consent was obtained from all patients.

### Data collection

We made a short clinical research form consisting of all variables that are described in the 2013 ACR/EULAR classification criteria for SSc, except anti-RNA polymerase III antibody, because this test was not routinely available at our institute. Two rheumatologists performed physical examinations, collected blood samples for autoantibodies related to SSc, and completed the clinical research form with cross-sectional data as follows: epidemiological characteristics including age, sex and duration of RP, clinical variables including proximal scleroderma, puffy finger, sclerodactyly, digital tip ulcer, fingertip pitting scar, telangiectasia, nailfold capillaries findings by nailfold capillaroscopy, pulmonary arterial hypertension and interstitial lung disease during follow-up. Pulmonary arterial hypertension was scored when it had been confirmed by cardiac catheterisation, which was performed in patients showing significantly elevated right ventricular systolic pressure on baseline echocardiography. Interstitial lung disease was defined when it had been detected on a chest X-ray or high-resolution computed tomography (HRCT). A chest X-ray was performed in all patients, but HRCT was conducted in only 49 of 124 participants and only 17 of 64 patients with RP who were suspected of interstitial lung disease based on simple chest X-ray studies. There were no significant differences in the rate of performing HRCT between patients reclassified as SSc versus those who were not reclassified. We also assessed the presence of SSc-related autoantibodies, including anti-centromere antibody or a centromere pattern seen on antinuclear antibody test, and anti-Scl-70 antibody in all patients. We counted clinical symptoms and radiological or laboratory results as positive items of the new classification criteria through three conditions: (1) physical examination at the time the patient provided informed consent, (2) review of systems and medical history taking, and (3) review of electronic medical records.

### Statistical analysis

All statistical analyses were conducted using the SPSS software (ver. 20.0 for Window; IBM Corp., Armonk, NY, USA). Levels of continuous variables were expressed as means ± standard deviations. Continuous variables between the two groups were compared using Mann–Whitney *U* tests, and non-continuous variables of age, sex and the presence of autoimmune disease, clinical manifestations and autoantibodies were assessed using a chi-square test. The odds ratio (OR) was assessed using a multivariate logistic regression test among variables with *P* values <0.05 in univariate analyses. For all statistical evaluations, *P* values <0.05 were considered to indicate statistical significance. We also applied Bonferroni correction to variables with statistical significance by making uncorrected *P* values doubled as Bonferroni-adjusted *P* values, since only two comparative analyses among three subgroups were needed in this study.

## Results

### Baseline characteristics of patients who had RP, but who had not previously been classified as SSc

Baseline characteristics are described in Table 1. The mean age was 50.9 years old and 58 patients were female. The mean RP duration was 5.3 years. Seventeen of 64 patients (26.5%), who presented with RP, but did not fulfill the 1980 ACR classification criteria, were newly classified as SSc by the 2013 ACR/EULAR classification criteria. Also, 33 of 64 patients (51.6%) had autoimmune diseases other than SSc, and satisfied the classification criteria for those autoimmune diseases [12-16]: 15 had Sjögren syndrome, 8 had systemic lupus erythematosus, 5 had mixed connective tissue disease, 3 had rheumatoid arthritis, and 2 had inflammatory myopathy. Puffy finger (53.1%) was the most frequently observed clinical feature followed by sclerodactyly (10.9%) and telangiectasia (10.9%). Moreover, 46 patients (71.8%) showed abnormal nailfold capillaries, 5 had interstitial lung disease, and 1 had pulmonary arterial hypertension. Antinuclear antibody was detected in 36 of 64 patients with RP (56.3%). Furthermore, a centromere pattern on immunofluorescence was seen in 19 patients (29.7%). Anti-centromere and anti-Scl-70 antibodies were found in 22 (34.4%) and 2 (3.1%) patients, respectively.

**Table 1:** **Patients’ characteristics, clinical manifestations and laboratory results in patients with Raynaud’s phenomenon**

**Variables**	**Patients with Raynaud’s phenomenon (N = 64)**
**Characteristics**	
Age (years old)	50.9 ± 14.4
Sex, female (N (%))	58 (90.6)
Raynaud’s phenomenon duration (years)	5.3 ± 7.7
**Newly classified to systemic sclerosis by the 2013 ACR/EULAR classification criteria (N (%))**	17 (26.5)
**Autoimmune disease accompanied (N (%))**	33 (51.6)
Sjögren syndrome	15 (23.4)
Systemic lupus erythematosus	8 (12.5)
Mixed connective tissue disease	5 (7.8)
Rheumatoid arthritis	3 (4.7)
Inflammatory myopathy	2 (3.1)
**Clinical manifestations (N (%))**	
Scleroderma (proximal)	0 (0)
Puffy finger	34 (53.1)
Sclerodactyly	7 (10.9)
Digital tip ulcer	2 (3.1)
Fingertip pitting scar	0 (0)
Telangiectasia	7 (10.9)
Abnormal nailfold capillaries	46 (71.8)
Pulmonary arterial hypertension	1 (1.6)
Interstitial lung disease	5 (7.8)
**Autoantibodies (N (%))**	
Antinuclear antibody (centromere)	19 (29.7)
Anti-centromere antibody	22 (34.4)
Anti-Scl-70 antibody	2 (3.1)

Values are expressed as N (%) or mean ± standard deviation. ACR, American College of Rheumatology; EULAR, The European League Against Rheumatism.

### Comparison of variables between patients who were versus those who were not reclassified as SSc according to the new classification criteria

We divided patients, who presented with RP and who had not been classified as SSc by the previous classification criteria, into two groups according to the 2013 ACR/EULAR classification criteria (17 patients were reclassified as SSc and 47 were not), and compared their variables. There were no significant differences in age, sex, RP duration or the presence of accompanying autoimmune diseases between the two groups. The mean 2013 ACR/EULAR classification criteria score in patients reclassified as SSc was 10.5, while that in patients not reclassified was 6.1 (Table 2). In particular, the reclassified patients showed increased frequencies of sclerodactyly, digital tip ulcer, telangiectasia, abnormal nailfold capillaries and the presence of anti-centromere antibody (Table 2). When we applied Bonferroni correction to the statistical analysis, variables with statistical significance still showed Bonferroni-adjusted *P* value <0.05.

**Table 2:** **Comparison of variables between patients who were or were not reclassified as systemic sclerosis according to the 2013 ACR/EULAR classification criteria for systemic sclerosis**

**Variables**	**Patients reclassified as systemic sclerosis (N = 17)**	**Patients not reclassified as systemic sclerosis (N = 47)**	***P*** **value**	***P*** **value** ^*^
**Characteristics**				
Age (years old)	48.9 ± 12.4	51.7 ± 15.1	NS	
Sex, female (N (%))	17 (100)	41 (87.2)	NS	
Raynaud’s phenomenon duration (years)	3.4 ± 2.4	6.1 ± 9.0	NS	
**The 2013 ACR/EULAR classification criteria score**	10.5 ± 1.8	6.1 ± 1.7	<0.001	<0.001
**Autoimmune disease accompanied (N (%))**	10 (58.8)	23 (48.9)	NS	
Sjögren syndrome	4 (23.5)	11 (223.4)	NS	
Systemic lupus erythematosus	4 (23.5)	4 (8.5)	NS	
Mixed connective tissue disease	2 (11.8)	3 (6.4)	NS	
Rheumatoid arthritis	2 (11.8)	1 (2.1)	NS	
Inflammatory myopathy	1 (5.9)	1 (2.1)	NS	
**Clinical manifestations (N (%))**				
Scleroderma (proximal)	0 (0)	0 (0)	NS	
Puffy finger	12 (70.6)	22 (46.8)	NS	
Sclerodactyly	6 (35.2)	1 (2.1)	<0.001	<0.001
Digital tip ulcer	2 (11.8)	0 (0)	0.017	0.034
Fingertip pitting scar	0 (0)	0 (0)	NS	
Telangiectasia	5 (29.4)	2 (4.3)	0.004	0.008
Abnormal nailfold capillaries	17 (100)	29 (61.7)	0.003	0.006
Pulmonary arterial hypertension	0 (0)	1 (2.1)	NS	
Interstitial lung disease^*^	2 (11.8)	3 (6.4)	NS	
**Autoantibodies (N (%))**				
Antinuclear antibody (centromere)	9 (52.9)	16 (34.0)	NS	
Anti-centromere antibody	11 (64.7)	11 (23.4)	0.002	0.004
Anti-Scl-70 antibody	0 (0)	2 (4.3)	NS	

Values are expressed as N (%) or mean ± standard deviation. *P* value^*^ = Bonferroni-adjusted *P* value among variables with statistical significance. ACR, American College of Rheumatology; EULAR, The European League Against Rheumatism; NS, not significant.

### Comparison of variables between patients who were newly classified as SSc and those who had been previously classified as SSc

When we applied the 2013 ACR/EULAR classification criteria to patients who had been classified as SSc by the 1980 ACR classification criteria, we found that all of the patients with SSc also met the new classification criteria. We compared variables between the newly classified and previously classified patients; the results are summarised in Table 3. There were no significant differences in age or sex between the two groups. The previously classified patients exhibited higher 2013 ACR/EULAR classification criteria scores than the newly classified ones (19.1 vs. 10.5, *P* <0.001). Nineteen (31.7%) of the previously classified patients presented with fingertip pitting scar, but none of the newly classified patients did. In addition, sclerodactyly and interstitial lung disease were more frequent in the previously classified patients, while telangiectasia was more frequent in the newly classified patients. The detection rates of antinuclear antibody (centromere) and anti-centromere antibody in the newly classified patients were higher than those in previously classified patients. However, anti-Scl-70 antibody was only detected in the previously classified patients. When we applied Bonferroni correction to the statistical analysis, variables with statistical significance still showed Bonferroni-adjusted *P* value <0.05.

**Table 3:** **Comparison of variables between patients who were newly classified as systemic sclerosis according to the 2013 ACR/EULAR classification criteria for systemic sclerosis and those who had been previously classified**

**Variables**	**Patients newly classified as systemic sclerosis (N = 17)**	**Patients previously classified as systemic sclerosis (N = 60)**	***P*** **value**	***P*** **value** ^*^
**Characteristics**				
Age (years old)	48.9 ± 12.4	51.1 ± 13.1	NS	
Sex, female (N (%))	17 (100)	52 (86.7)	NS	
**The 2013 ACR/EULAR classification criteria score**	10.5 ± 1.8	19.1 ± 5.1	<0.001	<0.001
**Clinical manifestations (N (%))**				
Scleroderma (proximal)	0 (0)	40 (66.7)	<0.001	<0.001
Puffy finger	12 (70.6)	27 (45.0)	NS	
Sclerodactyly	6 (35.2)	55 (91.7)	<0.001	<0.001
Digital tip ulcer	2 (11.8)	20 (33.3)	NS	
Fingertip pitting scar	0 (0)	19 (31.7)	0.008	0.016
Telangiectasia	5 (29.4)	5 (8.3)	0.022	0.044
Abnormal nailfold capillaries	17 (100)	56 (93.3)	NS	
Pulmonary arterial hypertension	0 (0)	3 (5.0)	NS	
Interstitial lung disease^*^	2 (11.8)	29 (48.3)	0.007	0.014
**Autoantibodies (N (%))**				
Antinuclear antibody (centromere)	9 (52.9)	12 (20.0)	0.007	0.014
Anti-centromere antibody	11 (64.7)	13 (21.6)	0.001	0.002
Anti-Scl-70 antibody	0 (0)	29 (48.3)	<0.001	<0.001

Values are expressed as N (%) or mean ± standard deviation. *P* value^*^ = Bonferroni-adjusted *P* value among variables with statistical significance. ACR, American College of Rheumatology; EULAR, The European League Against Rheumatism; NS, not significant.

### The predictive value of reclassification strategy

In multivariate logistic regression analyses of variables with significant differences between patients who were or were not newly classified (Table 2), the variables with independent predictive value were sclerodactyly (OR 60.025, *P* = 0.002), telangiectasia (OR 13.353, *P* = 0.030) and the presence of anti-centromere antibody (OR 11.168, *P* = 0.005) (Table 4).

**Table 4:** **The predictive values for the reclassification of systemic sclerosis in patients who showed Raynaud’s phenomenon and who had not been classified as systemic sclerosis**

**Variables**	**OR**	**95% confidence interval**
Sclerodactyly	60.025	4.311 - 835.753
Digital tip ulcer	NS	NS
Telangiectasia	13.353	1.292 - 137.961
Abnormal nailfold capillaries	NS	NS
Anti-centromere	11.168	2.076 - 60.073

OR, odds ratio; NS, not significant.

## Discussion

We enrolled patients who presented with RP, but who were not classified as SSc according to the previous classification criteria to assess the effectiveness of the new classification criteria for SSc and investigate potential clues to RP. For early classification of SSc, RP has several advantages in that RP and SSc have common immune-mediated aetiologies; RP is the sole self-reported symptom that occurs in the early phase of the disease, and it can precede typical fibrosis. In the present study, 17 of 64 patients (26.5%), who presented with RP, but did not fulfill the 1980 ACR classification criteria, were newly classified as SSc, when the 2013 ACR/EULAR classification criteria was used. Although RP is a clinical feature and sign of SSc and it can occur prior to fibrosis, the previous classification criteria missed the potential of SSc in a significant number of patients apparently without SSc who did present with RP. Thus, we anticipate a better opportunity to categorise and classify SSc in patients with RP using the 2013 ACR/EULAR classification criteria and applying it earlier.

Recently, several observational studies have used immunosuppressive agents, such as mycophenolate mofetil and rituximab, to delay or modify the disease course of SSc, but the therapeutic efficacies of these agents have not yet been fully validated [17-19]. However, considering the intractable and serious systemic complications of SSc, including pulmonary arterial hypertension and interstitial lung disease and the lack of proven predictive values for their development or exacerbation, we anticipate a ‘window of opportunity’ to classify SSc earlier [20]. Thus, we expect that the new classification criteria for SSc including RP could provide a chance to regularly monitor and follow up on major complications and not miss the appropriate time to initiate therapeutic trials for each systemic complication of SSc.

We found that patients, who were newly classified as SSc, had sclerodactyly, digital pitting scar, tip ulcer, telangiectasia, abnormal nailfold capillaries and anti-centromere antibody more frequently than those who were not reclassified. Furthermore, sclerodactyly, telangiectasia and the presence of anti-centromere antibody had independent predictive value for the reclassification of SSc in patients with RP; all of these variables are clinical and laboratory features that are usually observed in the early phase of SSc [1,11]. Moreover, they can be used to better monitor the more serious systemic complications of SSc. Thus, when patients visit the clinic, presenting with RP, we suggest that physicians should apply the new classification criteria for SSc, especially in patients who present with sclerodactyly or telangiectasia or who have anti-centromere antibody.

Furthermore, when we compared variables between the newly and previously classified patients, although sclerodactyly did not differ significantly between the two groups, telangiectasia and anti-centromere were significantly more frequent in the newly classified patients. Our results also verify the attainment of one of the goals of establishing the new classification criteria, namely, to overcome the low sensitivity of the previous system for detecting early-phase and limited types of SSc [11].

The strength of this study is that we first investigated the rate of the reclassification as SSc in patients who presented with RP, but did not fulfill the previous classification criteria. Furthermore, we obtained the independent predictive value for reclassifying patients with RP as SSc.

Our study also had several limitations. First, it was s cross-sectional study. Second, the severities of RP and other clinical manifestations were relatively high because our hospital is a tertiary institution. Third, we did not perform anti-RNA polymerase III test, which might have affected the rate of reclassification [11].

## Conclusions

Overall, 26.5% of the patients, who presented with RP, but who did not fulfill the 1980 ACR classification criteria, were newly classified as SSc according to the 2013 ACR/EULAR classification criteria. Sclerodactyly, telangiectasia, and the presence of anti-centromere antibody had independent predictive value for reclassifying patients with RP as SSc.

## Abbreviations

ACR: American College of Rheumatology
EULAR: The European League Against Rheumatism
HRCT: high-resolution computerized tomography
OR: odds ratio
RP: Raynaud’s phenomenon
SSc: systemic sclerosis
